# Interventions to Increase Leukocyte Testing during Treatment with Dimethyl Fumarate

**DOI:** 10.3390/ijerph181910312

**Published:** 2021-09-30

**Authors:** Paul A. Heidenreich, Shoutzu Lin, Parisa Gholami, Von R. Moore, Muriel L. Burk, Peter A. Glassman, Francesca E. Cunningham, Anju Sahay

**Affiliations:** 1 Veterans Affairs Palo Alto Health Care System, Palo Alto, CA 94304, USA; shoutzu.lin@va.gov (S.L.); parisa.gholami@va.gov (P.G.); anju.sahay@va.gov (A.S.); 2 Department of Medicine, Stanford University School of Medicine, Stanford, CA 94305, USA; 3 Veterans Affairs Center for Medication Safety Pharmacy Benefits Management Services, Hines, IL 60141, USA; von.moore@va.gov (V.R.M.); muriel.burk@va.gov (M.L.B.); fran.cunningham@va.gov (F.E.C.); 4 Veterans Affairs VA Pharmacy Benefits Management Services, Washington, DC 20004, USA; peter.glassman@va.gov; 5 Veterans Affairs Greater Los Angeles Healthcare System, Los Angeles, CA 90073, USA

**Keywords:** outcome assessment, health care, clinical pharmacy information systems, pharmacy services, multiple sclerosis, psoriasis, United States Department of Veterans Affairs

## Abstract

Dimethyl fumarate (DMF), a treatment for multiple sclerosis, may cause leukopenia and infection. Accordingly, periodic white blood cell (WBC) monitoring is recommended. We sought to evaluate the US Department of Veteran Affairs’ safety program which provides facilities with a list of patients prescribed DMF therapy without a documented white blood cell count (WBC). We identified 118 sites with patients treated with DMF from 1 January 2016 through 30 September 2016. Each site was asked if any of seven interventions were used to improve WBC monitoring (academic detailing, provider education without academic detailing, electronic clinical reminders, request for provider action plan, draft orders for WBC monitoring, patient mailings, and patient calls). The survey response rate was 78%. For the 92 responding sites (78%) included sites (1115 patients) the mean rate of WBC monitoring was 54%. In multivariate analysis, academic detailing increased the rate by 17% (95% CI 4 to 30%, *p* = 0.011) and provider education increased the rate by 9% (95% CI 0.6 to 18%, *p* = 0.037). The WBC monitoring rate increased by 3.8% for each additional intervention used (95% CI 1.2–6.4%, *p* = 0.005). Interventions focused on the physician, including academic detailing, were associated with improved WBC monitoring for patients at risk for leukopenia from DMF treatment.

## 1. Introduction

Dimethyl fumarate (DMF), a medication for treatment of multiple sclerosis and advanced psoriasis, can cause leukopenia which has been associated with infection including fatal progressive multifocal leukoencephalopathy [1,2,3,4,5,6]. Accordingly, white blood cell (WBC) monitoring is recommended for those on DMF therapy so treatment can be adjusted or discontinued if severe leukopenia occurs. In one study most patients had at least a slight drop in the lymphocyte count [7]. Sixty percent dropped from a mean of 2.1 to 1.9 × 10^9^ cells/L (small), 9.4% had a moderate drop (1.7 to 0.9 × 10^9^ cells/L) and 2.1% had a severe drop (1.7 to 0.5 ×10^9^ cells/L) [7]. A threshold of lymphocyte count at which infection risk rises markedly is not clear. The rare cases of progressive multifocal leukoencephalopathy that are associated with DMF use were observed during moderate to severe, prolonged lymphopenia [8].

To improve the safety of DMF use, the US Department of Veteran Affairs’ (VA) has included this medication as part of its national Medication Use Evaluation Tracker (MUET) safety program. [9] The DMF MUET safety program identifies potentially at-risk patients filling prescriptions for DMF therapy either without a documented VA WBC count follow-up within 3 months of a released prescription, or with a dangerously low WBC. MUET provides pharmacists at VA Health Care Systems (sites) with secured lists of patients who meet these at-risk criteria, and the VA pharmacists then intervene at their discretion. Thus, different interventions are used by different VA sites. Commonly used interventions to address safety monitoring include provider education including academic detailing, patient education and computerized clinical reminders [9,10,11].

The purpose of this study was to evaluate participation in the DMF MUET safety program, determine use of different interventions to improve WBC monitoring, and determine if any intervention was associated with greater WBC monitoring.

## 2. Materials and Methods

VA Sites: We identified VA sites with patients who filled prescriptions for DMF during the target time period (1 January 2016–30 September 2016). This time period was selected to obtain a study size that should be adequate to detect a 10% difference in WBC monitoring between interventions. Sites were made up of at least one hospital with one or more outpatient clinics which may be dispersed geographically. Site characteristics were determined using linkage with survey data from the American Hospital Association to determine membership in the Council of Teaching Hospitals (COTH) and the presence of Accredited Graduate Medical Education (AGME) programs.

MUET Program: Patients were identified if they were on active DMF treatment (defined as at least one filled prescription) during a fiscal quarter with at least one of two criteria: (1) Absence of WBC count during the 90 days following a DMF prescription. (2) Most recent WBC <2000/mm^3^ within 90 days of a DMF prescription [9].

Data were obtained from the VA Corporate Data Warehouse outpatient prescription data and were updated monthly. Laboratory data were updated daily. A list of at-risk patients was sent to each site on a quarterly basis. Each site was asked to provide a response for each patient (providing a reason for not intervening, or state that lab testing was ordered or that the medication dose was adjusted or discontinued). The pharmacy service at each site could decide if and how they wished to intervene on any of any patient on the list.

Pharmacy-Led Interventions: VA Pharmacy leadership identified seven interventions known to be used by local pharmacists: (1) academic detailing by pharmacists, (2) provider education without academic detailing, (3) electronic clinical reminders, (4) draft orders for WBC testing, (5) request for care plan from the provider, (6) patient calls, and (7) patient mailings. Pharmacists were surveyed to determine if any, and which of these seven interventions were used at their site. If more than one pharmacist responded from a given site, we used the response from the pharmacist in the highest leadership position.

White Blood Cell Count Monitoring: The primary outcome was WBC count monitoring in the 90 days after a filled prescription for DMF at the site level expressed as a percent of all patients with a filled DMF prescription.

Statistical Analyses: We compared means of site rates of 90-day WBC monitoring for sites grouped by the use of the seven interventions described above (site level analysis). We weighted all analyses using the number of patients with a filled prescription at each site. To determine the interventions independently associated with higher WBC monitoring we performed a forward stepwise regression analysis where interventions were entered sequentially (in order of statistical significance from unadjusted analyses) and then removed if the *p* value remained >0.10. A *p*-value < 0.05 was considered statistically significant for this exploratory analysis.

The survey and primary analysis were performed as part of VA operations (quality improvement) to determine the efficacy of the MUET program. Sites that did not respond to the survey (missing data) were not included in the analysis. Institutional Review Board (IRB) approval (Stanford University) was obtained to perform additional (secondary) analyses for publication. The manuscript adheres to the STROBE recommendations for cohort studies (supplementary material).

## 3. Results

We identified 118 VA sites treating patients with DMF. The site response rate for the survey assessing interventions was 78% (92 of 118 sites). The 92 sites (1115 patients) were located more in the South (41%) and Midwest (24%), compared with the Northeast (20%) and West (15%). Data regarding academic status from the American Hospital Association were available for 89 of 92 sites (97%), of which 34 (38%) were members of the Council of Teaching Hospitals (COTH) and 68 (76%) had Accredited Graduate Medical Education (ACGME) programs. There were no significant differences in characteristics between responding and non-responding sites.

### 3.1. Use of the Dimethyl Fumarate MUET Safety Program

Among the 92 responding sites, 51 (55%) sites reported participating in the DMF MUET safety program of the Medication Use Evaluation Tracker (MUET). Of these, the safety program was described as very useful by 20 (39%), useful by 27 (53%) and not useful by 4 (8%) sites.

### 3.2. Interventions and WBC Monitoring

Among those sites participating in the DMF MUET safety program, the mean rate of WBC monitoring was 54%. Monitoring was greater in those sites describing the DMF MUET safety program participation as useful (55% ± 15%) compared to those saying that participation was not useful (37%± 18%, *p* = 0.03). Table 1 describes the use of the seven interventions and the rates of WBC monitoring for sites using those interventions. The interventions with the greatest impact on WBC monitoring were academic detailing, draft orders for WBC testing, provider education and request for a provider management plan.

### 3.3. Adjusted Analyses

In stepwise multivariate analysis, only academic detailing and provider education remained significantly associated with WBC monitoring. In this model, doing neither of these interventions was associated with a WBC monitoring rate of 46%. Academic detailing increased the rate by 17% (95% confidence interval 4 to 30%, *p* = 0.011) and provider education increased the rate by 9% (95% CI 0.6 to 18%, *p* = 0.037).

### 3.4. Number of Interventions

**Figure 1:**
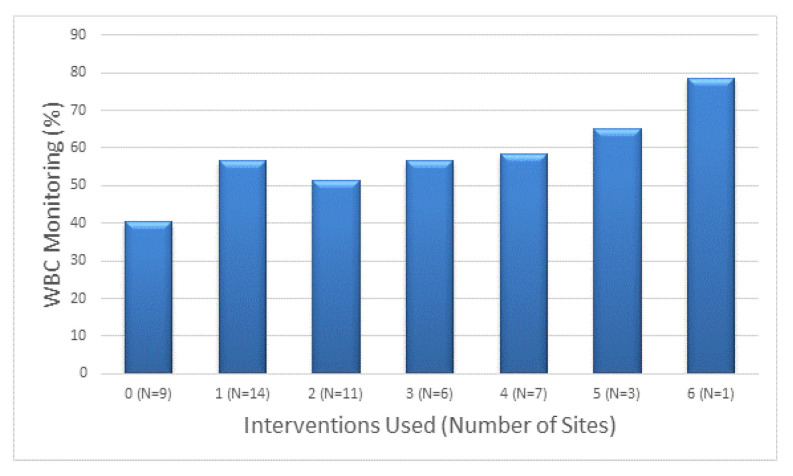
The rate of WBC monitoring is shown for sites using increasing number of interventions. The monitoring rate increased with greater use of interventions (*p* = 0.05 for trend). When WBC rate was considered continuous the trend was more significant (*p* = 0.005), 3.8% increase for each additional intervention (95% CI 1.2–6.4).

Overall, 82% of participating sites (42/51) reported using at least one intervention, 55% (28/51) used two or more and 33% (17/51) used three or more interventions. Monitoring of WBC increased with an increasing number of interventions used (Figure 1). When WBC monitoring rate was examined as a continuous variable, the WBC monitoring rate increased by 3.8% for each additional intervention (95% CI 1.2–6.4%, *p* = 0.005).

### 3.5. Barriers to Participation

The reasons for lack of any participation or discontinuation in the DMF MUET safety program are shown in Table 2 for the 41 sites that did not participate. Most (75%, 31/41) provided at least one reason for lack of participation. The most common reasons were lacking enough patients taking DMF to make this worthwhile, a concern that participating will be too time consuming, and the lack of real time data. Among sites those that discontinued their involvement with the DMF MUET safety program, 36% felt their providers were educated adequately.

## 4. Discussion

Our study examined site participation in the VA’s Medication Use Evaluation Tracker (MUET) safety program [9] that identifies patients taking dimethyl fumarate (DMF) who were not monitored with a white blood cell (WBC) count. We found that VA sites participating in the DMF MUET safety program use a variety of interventions to improve WBC monitoring for patients taking DMF. Sites were free to select which interventions to implement to improve safety monitoring of patients taking DMF. The most common interventions used were electronic reminders, provider education and a request for a provider management plan. We took advantage of this natural experiment to evaluate the association of these interventions with higher WBC monitoring.

The MUET software program was designed to improve medication safety throughout the VA’s network of 147 medical centers [9]. MUET initiatives are managed by the VA Center for Medication Safety (VAMedSAFE) which aims to reduce inappropriate prescribing and inadequate monitoring with the goal of reducing medication-related adverse effects. The VAMedSAFE center sends a list of at-risk veterans, based on inappropriate prescriptions or inadequate testing, to a shared electronic site where locally designated personnel (typically pharmacists) can review it [9]. Each site can then decide on which intervention, if any, to apply. The MUET lists are updated periodically to enable tracking and reporting for surveillance and quality-improvement purposes. In a pilot project, the MUET program was found effective in promoting improved appropriate prescribing of erythropoiesis-stimulating agents (ESAs) and enhanced laboratory monitoring of ESA-treated patients. In response to the list, many patients had their ESA discontinued or had their doses reduced [9].

We identified two of seven pharmacy-led interventions that were significantly and independently associated with higher WBC monitoring of patients taking DMF. After adjustment, WBC monitoring increased above the baseline of 46% by 9% if provider education (without academic detailing) was used and by 17% if academic detailing was used. While other interventions did not reach statistical significance at the *p* < 0.05 level, there were trends toward improved monitoring with most interventions. The more interventions used, the higher the (WBC) monitoring with a 3.8% increase in monitoring for each additional intervention used.

Academic detailing had been successful in multiple care settings [12,13,14], though the specific methods of successful detailing are unclear [10]. In a review of external change agents to promote quality improvement, Alagoz found that academic detailing was the most commonly used strategy [12]. Typically, a pharmacist was used as the academic detailer though some programs used physicians or nurses. Almost all the academic detailing interventions used audit and feedback though studies that included individualized follow-up demonstrated greater effects. Academic detailing programs that incorporated practice facilitation or coaching were more likely to demonstrate an improvement in the primary outcome [12]. In a review of 106 academic detailing studies designed to improve medication management, the mean number of visits per provider was 2.8 ± 5.4 a mean 3.5 ± 2.2 months apart [10]. The most common duration (mode) of an academic detailing visit was one hour. Within the VA, academic detailing of VA pharmacists to primary care providers has been demonstrated to reduce potentially inappropriate medications in a pre-post analysis [15]. VA academic detailing has led to a 3.4% decrease in opioid use in one VA network, and an almost 2% decrease in concomitant opioid and benzodiazepine prescribing in another VA network [16].

Medication alert messages at the point of Computerized Provider Order Entry (CPOE) have been evaluated for reducing potentially inappropriate medications [17,18] in elderly Veterans. In a pre-post design, electronic alerting was not associated with a reduction in potentially inappropriate medication use, though a significant reduction was found when limited to the top 10 most common newly prescribed potentially inappropriate medications, 9.0% to 8.3% (*p* = 0.016) [19]. In randomized trials, electronic medication alerts have been successful in modifying prescribing patterns, but the effect is modest often requiring a large sample size to demonstrate significance [20,21]. This is consistent with our study’s finding that electronic reminders appeared promising but not as strong as academic detailing. The limited effect may be due to a lack of coordination between nurses and providers, using the reminders while not with the patient, and not having a link to the recommended action [11].

One reason for not participating in the safety program was the lack of real time data. Thus, use of a dashboard to disseminate patient information may increase participation. While not specifically asked in the survey, several of the sites not participating were known to have access to a local (own site) dashboard.

Other interventions not used by the VA facilities may be effective. A personal health record was examined in a randomized trial of older adults [22]. Patients aged 65 who also used computers were randomized to a PHR to determine the impact on medication reconciliation behaviors, and medication management problems. Only 16% of those randomized to the PHR used it frequently. However, the PHR was effective in reducing use of multiple non-steroidal anti-inflammatory drugs-the most common warning generated by the system. Compared with low/non-users, high users reported significantly more changes in medication use and improved medication reconciliation behaviors [22].

Limitations: There are several potential limitations of the evaluation. Sites were not randomized to the different interventions, and it is possible that differences in sites (e.g., attitudes towards safety) may have led to both the use of interventions and improved WBC monitoring. It is possible that some patients may have had lab testing at non-VA sites. Such unobserved testing would have biased the results towards the null, making it more difficult to see significant associations. Variation in delivery of the strategies is likely to have occurred across sites (e.g., different ways and persistence in delivering provider education or academic detailing). Large variation in delivery would make it more difficult to see significant effects so our positive findings for certain strategies suggests they may be robust to variation in adaption. Our analysis also occurred before the implementation of a pharmacy practice program for each medical home (Patient Aligned Care Team) [16]. With such programs, pharmacists have prescriptive authority and can assume medical management for targeted conditions. Finally, VA sites may be more amenable to implementation of quality improvement interventions than non-VA sites.

## 5. Conclusions

In summary, we found that provider education, particularly through pharmacist-led academic detailing was associated with improved WBC monitoring for patients treated with dimethyl fumarate. Greater use of interventions by facilities was associated with greater WBC monitoring. The study demonstrates how pharmacists can respond effectively to receiving lists of patients at risk. Such lists are created through system-level tracking of medication use and lab monitoring. The wider implementation of provider education to improve lab monitoring for medication safety should be studied.

## Figures and Tables

**Table 1:** Use and Effectiveness of Interventions to Improve WBC Monitoring Among 51 Participating Sites. (CI = Confidence Interval).

Intervention	Site Use N (%, 95% CI)	Mean Rate of WBC Monitoring (% of Patients)		*p*-Value
		Using Intervention	Not Using Intervention	
Electronic Reminders	30 (59%, 45%–72%)	57 ± 16	50 ± 16	0.13
Provider Education	27 (53%, 39%–67%)	59 ± 13	47 ± 18	0.008
Provider Request for Management Plan	26 (52%, 37%–65%)	58 ± 17	50 ± 14	0.07
Patient Calls	7 (14%, 4%–23%)	57 ± 14	53 ± 17	0.33
Academic Detailing	6 (12%, 3%–21%)	73 ± 9	52 ± 15	0.003
Draft Orders for WBC	5 (10%, 2%–18%)	66 ± 11	53 ± 16	0.09
Patient Mailing	2 (4%, 0%–9%)	44 ± 9	53 ± 17	0.47

**Table 2:** Reasons for Lack of Participation in MUET Safety Program.

Sites Previously Participating (N = 14)	N (%) *
Not enough patients to make it worthwhile	9/14 (64%)
Providers adequately educated	5/14 (36%)
Not a high priority safety issue	1/14 (7%)
Any reason provided	12/14 (86%)
**Sites that Never Participated (N = 27)**	
Too time consuming	8/27 (30%)
Not enough patients to make it worthwhile	6/27 (22%)
Not real time data	4/27 (15%)
Need help from other services to implement	2/27 (7%)
Not a high priority safety issue	2/27 (7%)
Not appropriate work for a pharmacist	1/27 (4%)
Information Technology (IT) limitation	1/27 (4%)
Any reason provided	19/27 (70%)

* More than one reason could be provided for each category.

## Supplementary Materials

The following are available online at https://www.mdpi.com/article/10.3390/ijerph181910312/s1. Supplementary Materials title: STROBE Statement—Checklist of items that should be included in reports of *cohort studies*.
